# Methylation analysis of *Klebsiella pneumoniae* from Portuguese hospitals

**DOI:** 10.1038/s41598-021-85724-2

**Published:** 2021-03-22

**Authors:** Anton Spadar, João Perdigão, Jody Phelan, James Charleston, Ana Modesto, Rita Elias, Paola Florez de Sessions, Martin L. Hibberd, Susana Campino, Aida Duarte, Taane G. Clark

**Affiliations:** 1 Faculty of Infectious and Tropical Diseases, London School of Hygiene and Tropical Medicine, London, UK; 2 Research Institute for Medicines (iMed.ULisboa), Faculdade de Farmácia, Universidade de Lisboa, Lisboa, Portugal; 3 Genome Institute Singapore, Singapore, Singapore; 4 Faculdade de Farmácia, Universidade de Lisboa, Lisboa, Portugal; 5 Centro de Investigação Interdisciplinar Egas Moniz, Instituto Universitário Egas Moniz, Almada, Portugal; 6 Faculty of Epidemiology and Population Health, London School of Hygiene and Tropical Medicine, London, UK; 7 Department of Infection Biology, London School of Hygiene and Tropical Medicine, Keppel Street, London, WC1E 7HT UK

**Keywords:** Epigenomics, Antimicrobial resistance, Bacterial genomics

## Abstract

*Klebsiella pneumoniae* is an important nosocomial infectious agent with a high antimicrobial resistance (AMR) burden. The application of long read sequencing technologies is providing insights into bacterial chromosomal and putative extra-chromosomal genetic elements (PEGEs) associated with AMR, but also epigenetic DNA methylation, which is thought to play a role in cleavage of foreign DNA and expression regulation. Here, we apply the PacBio sequencing platform to eight Portuguese hospital isolates, including one carbapenemase producing isolate, to identify methylation motifs. The resulting assembled chromosomes were between 5.2 and 5.5Mbp in length, and twenty-six PEGEs were found. Four of our eight samples carry *bla*_CTX-M-15_, a dominant Extended Spectrum Beta Lactamase in Europe. We identified methylation motifs that control Restriction–Modification systems, including GATC of the DNA adenine methylase (Dam), which methylates N6-methyladenine (m6A) across all our *K. pneumoniae* assemblies. There was a consistent lack of methylation by Dam of the GATC motif downstream of two genes: *fosA*, a locus associated with low level fosfomycin resistance, and *tnpB* transposase on IncFIB(K) plasmids. Overall, we have constructed eight high quality reference genomes of *K. pneumoniae*, with insights into horizontal gene transfer and methylation m6A motifs.

## Introduction

*Klebsiella pneumoniae* (*Kp*) are Gram-negative bacteria that are found in the normal flora of the mouth, intestines, skin and faeces, but in other parts of the body, such as the lungs, can cause severe morbidity with a diverse disease spectrum that can culminate in complicated invasive infections. This pathogen is increasingly recognized as an important etiological agent of healthcare associated infections. *Kp* has also been identified as a key route of introduction and dissemination of antimicrobial resistance (AMR) genes into other clinically significant pathogens^1,2^. In Europe, *Kp* with resistance to fluoroquinolones and carbapenems, and third-generation cephalosporins has been increasing, leading to reduced treatment options^3^.

The genome sequencing of *Kp c*linical isolates can provide insights into AMR, but also epigenetic information superimposed over nucleotide sequences^4^. The formation of epigenetic lineages enables the adaptation of bacterial populations to harsh or changing environments and modulates the interaction of pathogens with their eukaryotic hosts^5–7^. Epigenetic signals control DNA–protein interactions and can cause phenotypic change in the absence of mutation^8^. A common mechanism of epigenetic signalling is DNA methylation by orphan methyltransferases (MTases) such as Dam, which have roles in chromosome replication and segregation, nucleoid organization, cell cycle control, and DNA repair^4,8,9^. DNA methylation is also the key element of Restriction–Modification (R–M) systems that not only provide defence against foreign DNA, but also encourage bacterial evolution by driving the persistence of plasmids and other mobile genetic elements^10,11^.

Single Molecule, Real-Time (SMRT) platforms detect DNA modifications by measuring variation in the polymerase kinetics of DNA base incorporation during sequencing. The approach has the ability to detect genome-wide MTase N6-methyladenine (m6A) and N4-methylcytosine (m4C) target motifs at coverage levels recommended for assembly, and reveal phase variation of related genes^12^. DNA adenine methylase (Dam), which methylates m6A in the GATC sequence, plays a key role in DNA mismatch repair, as well as in bacterial virulence and gene expression, including in some strains of *Kp*^10,13–15^. R–M systems have also been observed on bacterial plasmids where they may contribute to their maintenance^11^. *Kp* is known to have type I and type II R–M systems^16^. The two systems have different mechanics and their distinct motif types have been reviewed elsewhere^4,8^. Briefly, type I R–M systems consist of specificity, modification and restriction subunits. The first two subunits are usually located together on a chromosome and are under the control of the same promoter^17^. The restriction subunit in the type I R–M system recognises long bipartite motifs, such as GGCAN_8_TCG. While not part of complete type II R–M, Dam is the most common MTase in Gamma-proteobacteria and it recognises palindromic 5′-GATC-3′ motifs^4,8^.

Here we applied SMRT sequencing to eight *Kp* isolates from Portugal with antibiotic susceptibility phenotyping to characterise the bacterial epigenome and explore the relationship between methylation and AMR. We focused on methylation, including around AMR genes, and on differences in the abundance of R–M recognition motifs on *Kp* chromosomal and mobile genetic elements. The abundance of some target methylation motifs was different between chromosomes and plasmids, especially the GATC motifs methylated by orphan MTase Dam. We also found that a GATC motif immediately downstream of the *fosA* gene, which confers low level fosfomycin resistance^18^, is consistently unmethylated in our samples. Isolates that had the *tnpB* transposase gene^19^ on the IncFIB(K) plasmid also consistently lacked methylation immediately downstream of this gene.

## Results

### Genome assemblies and phylogeny

**Figure 1:**
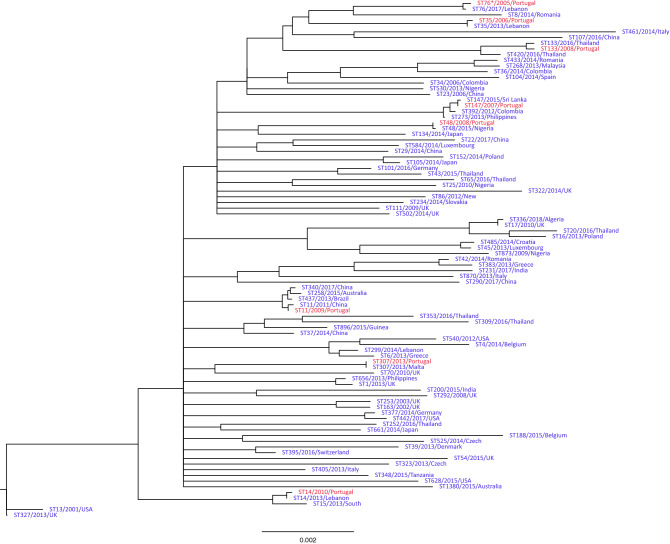
Unrooted maximum likelihood phylogenetic tree of common 83 *Kp* sequence types (blue) and the 8 isolates from our study (red). Edges with bootstrap support below 75 (based on 5000 bootstraps) were collapsed. Scale is nucleotide substitutions per site. Tip labels are sequence type, collection year and country.

Eight *Kp* isolates with different multi-locus sequence types (MLSTs)^20^ were sourced from two hospitals in Lisbon, Portugal, between 2005 and 2013, and sequenced on the PacBio RSII technology (Table 1). The assembled chromosomes were between 5.2 and 5.5 Mbp in length and had GC content values between 57.2 and 57.7%. By contrast, 26 putative extra-chromosomal genetic elements (PEGEs) ranged in length between 3.6 and 284.2 Kbp and did not segregate into clusters based on sequence length. The PEGEs have a GC content between 41.4 and 54.1%, except for an outlying 9.9 Kbp plasmid with 60.1%. This outlying plasmid had 100% coverage and 93% identity to several plasmids of Gram-negative bacteria (e.g. CP027616.1, CP023430.1) (Table 1). To put our samples in a broader context we constructed a maximum likelihood phylogenetic tree based on the alignment of seven MLST informative and nineteen core genome loci, which contained 83 representative *Kp* MLST groups sourced from the NCBI database (Fig. 1)^21^. All our isolates were the nearest neighbour of isolates with the same MLST, but there was varying heterogeneity in genetic distance between the samples from same MLST, resulting from using 19 additional loci that could differentiate geographical and temporal differences. For example, the nearest neighbour to our Kp3860 isolate (ST307) was one collected in Malta in the same year with an identical MLST and no sequence differences across the genes analysed. On the other hand, our Kp2209 isolate had some divergence from another ST133 sample isolated in Thailand eight years earlier.

**Table 1:** *Klebsiella pneumoniae* assemblies generated for the analysis.

ID	Hospital	Isolation Year	Fold coverage	N50 Mbps	Busco** complete /fragmented (%)	Non-chromosomal Contigs > 1kbps	MLST-type	K locus type	O locus type
Kp1363	HSAC	2005	197	5.34	89.4/7.3	1	76***	KL10	O3/O3a
Kp1208	HSM*	2006	130	5.37	98.5/0.2	2	35	KL22	O1v1
Kp1264	HSM	2007	131	5.23	97.5/0.9	4	147	KL64	O2v1
Kp1675	HSAC	2008	428	4.02	98.9/0.2	4	48	KL62	O1v1
Kp2209	HSM*	2008	504	5.37	98.5/0.5	4	133	KL116	O1v1
Kp2564	HSM	2009	36	5.39	98.7/0.2	6	11	KL111	O3b
Kp2958	HSM	2010	187	5.48	97.5/1.1	3	14	KL2	O1v1
Kp3860	HSM	2013	241	5.39	97.3/1.1	2	307	KL102	O2v1

All isolates sourced from blood, except * from urine; HSM Hospital de Santa Maria; HSAC Hospital St Antonio dos Capuchos; MLST sequence type; ** Busco score is based on genes set enterobacterales_odb10; *** Kp1363 matches six out of seven ST76 alleles; N50 is defined as the sequence length of the shortest contig at 50% of the total genome length.

### Antimicrobial resistance loci

Consistent with their Portuguese origin, four of our eight samples carry *bla*_CTX-M-15_, a dominant extra-spectrum beta-lactamase in Europe and a source of resistance to third-generation cephalosporins^22^. Two isolates, Kp1675 and Kp1264, carry the gene on a chromosomal region flanked by Tn2 (Tn3 family) and IS26 (family IS6) mobile genetic elements. Two further isolates, Kp3860 and Kp2209, carry *bla*_CTX-M-15_ gene on IncFIB(K) plasmids. Finally, in addition to chromosomal *bla*_CTX-M-15_, Kp1264 also carries it on IncFIA(HI1) plasmid which belongs to the same incompatibility group as IncFIB(K)^23^. All five *bla*_CTX-M-15_ fragments (4 isolates) are embedded in the 9500 nt sequence that is near identical between samples (> 99.98% similarity), though Kp2209 has only 50% coverage compared to 93% coverage for the other sequences. This 9500 nt sequence also carries quinolone and aminoglycoside resistance genes. In three isolates (Kp1675, Kp1264 and Kp3860), the region containing *bla*_CTX-M-15_ also contains *bla*_OXA-1_, which is a penicillinase and a major correlate of resistance to piperacillin/tazobactam and co-amoxiclav in *Kp* and *E. coli*, and is commonly associated with co-carriage of *aac(6′)-Ib-cr*, which restricts aminoglycoside and fluoroquinolone treatment options^24,25^. Only the Kp2564 isolate bore a KPC-3 carbapenemase coding gene (*bla*_KPC-3_), which is located on an IncFIA plasmid. This carbapenemase is thought to have originated in clonal group 258 (CG258), to which this sample belongs^26^. KPC-3 has now spread across *Kp* MLSTs globally, including in Portugal^27^, where its prevalence has increased dramatically over the last decade^28^. All samples were tested for susceptibility to major anti-microbials (Table S1). Sample Kp2564 was the only one resistant to imipenem, which is consistent with presence of *bla*_KPC-3_. Of all samples only Kp1264 appeared resistant to fosfomycin.

### Methylation analysis

Seven of the eight samples had sufficient quality and coverage to perform DNA methylation analysis using the SMRT portal. For the other sample (Kp2564), we successfully identified methylation motifs, but low sequencing coverage meant that for non-GATC motifs the detection of motifs methylation state was unreliable. After comparing the generality of motifs versus the frequency of motif methylation (Figure S1) for each isolate, we removed the motifs for which less than 60% of motif occurrences were methylated. For Kp2564 we focused only on GATC, AACN_5_RTGC and GCAYN_5_GTT motifs, the latter two being part of same type I bipartite recognition sequence. While our raw results did contain four m4C modifications, the highest modification rate was 17% and therefore omitted from further analysis.

All seven high quality assemblies contained a GATC recognition motif of the Dam based type R–M system. A total of ten type I R–M system motifs were found. For each of these ten we identified specific MTases (Table 2) by comparing their specificity subunits to those in the REBASE database^16^. For all but one specificity subunit, REBASE has 100% matches of the DNA target recognition domain. The recognition sequence motif on plasmid_5 of Kp2564 was the exception with 82% highest amino acid identity in the target domain. For Kp1363, SMRT Analysis returned four motifs (CGACCN_4_TGG, CGAYDN_4_TGG, CCAN_4_GATCA and CCAN_5_RTCG) without partner motif strings. The first and second motifs are imperfect reverse complements of the fourth. The third motif is likely to be recognised by the same specificity subunit, but may show low methylation (60%) due to the presence of a type II GATC motif within the type I motif. Given the highly similar number of sites (2336 for the first three motifs versus 2311 for the fourth), the similarity of motifs and only two specificity proteins in the assembly, we suspect that the four motifs are recognised by the same specificity subunit. A problematic sequence was GAAAN_6_GGG in Kp3860 for which the SMRT Analysis pipeline did not return a partner motif, nor plausible reverse complement. The assembly has one specificity protein, but has no exact match to known recognition sequences in REBASE, with the closest being GAGN_6_GGG from an *E. coli* specificity subunit (S.Eco77I), with identity 36% (e-value 7e-72).

**Table 2:** List of high-quality *m6A* motifs.

Assembly	Main motif/partner motif	Modified fraction	Number of motifs	Specificity subunit
Kp1208	GATC	98%	63,002	
Kp1208	TGAYN6TTTG/ CAAAN6RTCA	96%/88%	463	chr: 557,963–561,058
Kp1264	CCAGN7RTTC/ GAAYN7CTGG	98%/94%	347	chr: 1,565,436–1,567,199
Kp1264	GATC	98%	61,076	
Kp1264	GGCAN8TCG/ CGAN8TGCC	98%/91%	1042	chr: 1,638,656–1,639,996
Kp1363	CCAN4GATCA	60%	447	plasmid_1: 100,843–102,264
Kp1363	CCAN5RTCG	98%	2313	plasmid_1: 100,843–102,264
Kp1363	CGACCN4TGG	98%	276	plasmid_1: 100,843–102,264
Kp1363	CGAYDN4TGG	98%	1613	plasmid_1: 100,843–102,264
Kp1363	GAAAYN8TCG/ CGAN8RTTTC	97%/96%	459	chr: 1,946,700–1,947,668
Kp1363	GATC	99%	61,266	
Kp1675	GATC	99%	64,248	
Kp2209	GATC	99%	64,264	
Kp2209	GATGN6TTG/ CAAN6CATC	99%/99%	1029	plasmid_1: 2570–3844
Kp2564	AACN5RTGC/ GCAYN5GTT	26%/26%	998	plasmid_3: 94,898–96,112
Kp2564	GATC	36%	63,282	
Kp2958	ACAN8TGAC/ GTCAN8TGT	98%/96%	319	chr: 4,378,992–4,379,303
Kp2958	AGCN5CTTC/GAAGN5GCT	100%/98%	1024	chr: 1,411,097–1,412,662
Kp2958	GATC	98%	64,066	
Kp3860	GAAAN6GGG	97%	586	chr: 1,856,401–1,857,603
Kp3860	GATC	99%	63,388	

Only the GATC motif was present in multiple isolates, consistent with widespread presence of DNA adenine methylase among Gammaproteobacteria. Kp2564 has low methylation rates due to low sequencing coverage.

**Figure 2:**
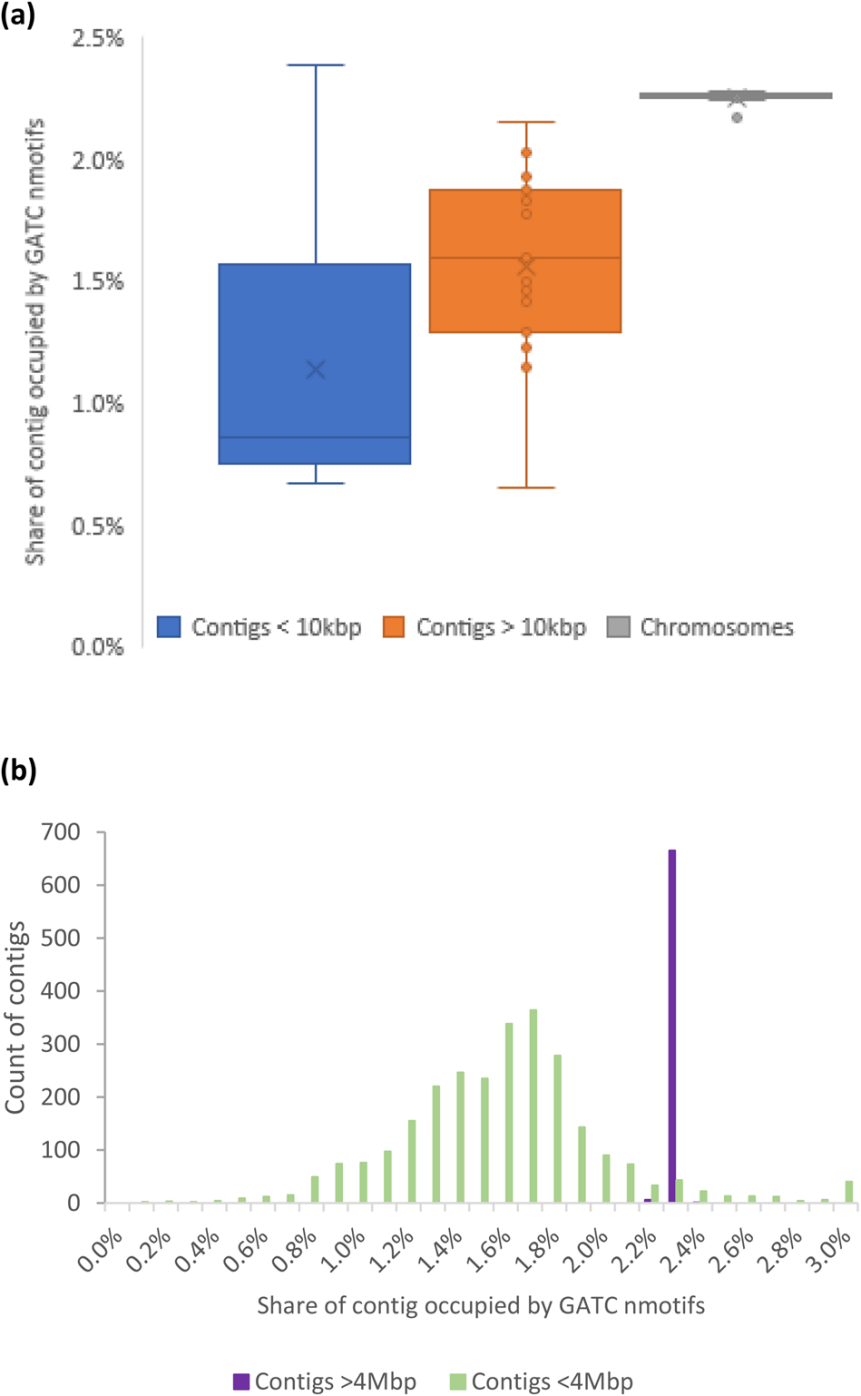
(**a**) The share of genomic elements occupied by a GATC motif in the assemblies in Table 1. The largest non-chromosome contig, 284kbp, has abundance ratio of 1.8%. (**b**) GATC abundance in 673 high quality assemblies of *Kp* taken from NCBI database limiting sequences to those that have N50 in excess of 5Mbp. The mean chromosomal GATC abundance in (**a**) and (**b**) is the same.

Due to a lack of a corresponding restriction enzyme in *Kp,* the GATC motif is not involved in the defence against foreign DNA^8^. To assess if this is reflected in the number of motifs on the *Kp* chromosome and PEGEs, we examined the relative abundance of GATC motifs, as measured by the ratio of total length of observed motifs to those of genomic sequence. For the GATC motif, there was much greater abundance on the chromosomes compared to PEGEs (Wilcoxon *P* < 3 × 10^−6^) (Table S2, Fig. 2a). We found the same result in 673 high quality *Kp* assemblies from the NCBI RefSeq database (all N50s > 4.5Mbp; Fig. 2b), where the GATC abundance on the chromosome (mean 2.25%; standard deviation 0.017%) was greater than on PEGEs (mean 1.52%; std. dev. 0.088%) (Wilcoxon *P* < 0.001). We also investigated the abundance of type I R–M system motifs (i.e. non-GATC) by comparing the share of chromosomes occupied by the motifs that are recognised by assembly’s MTases versus the share occupied by recognition sequences from other assemblies. There was no strong difference in the abundance of type I recognition motifs on chromosomes or between chromosomes and PEGEs (Wilcoxon *P* > 0.230). Similarly, there was no strong difference between PEGEs that were recognised by their own assembly’s R–M system compared to those that were not recognised (Wilcoxon *P* = 0.187).

### Unmethylated motifs

**Figure 3:**
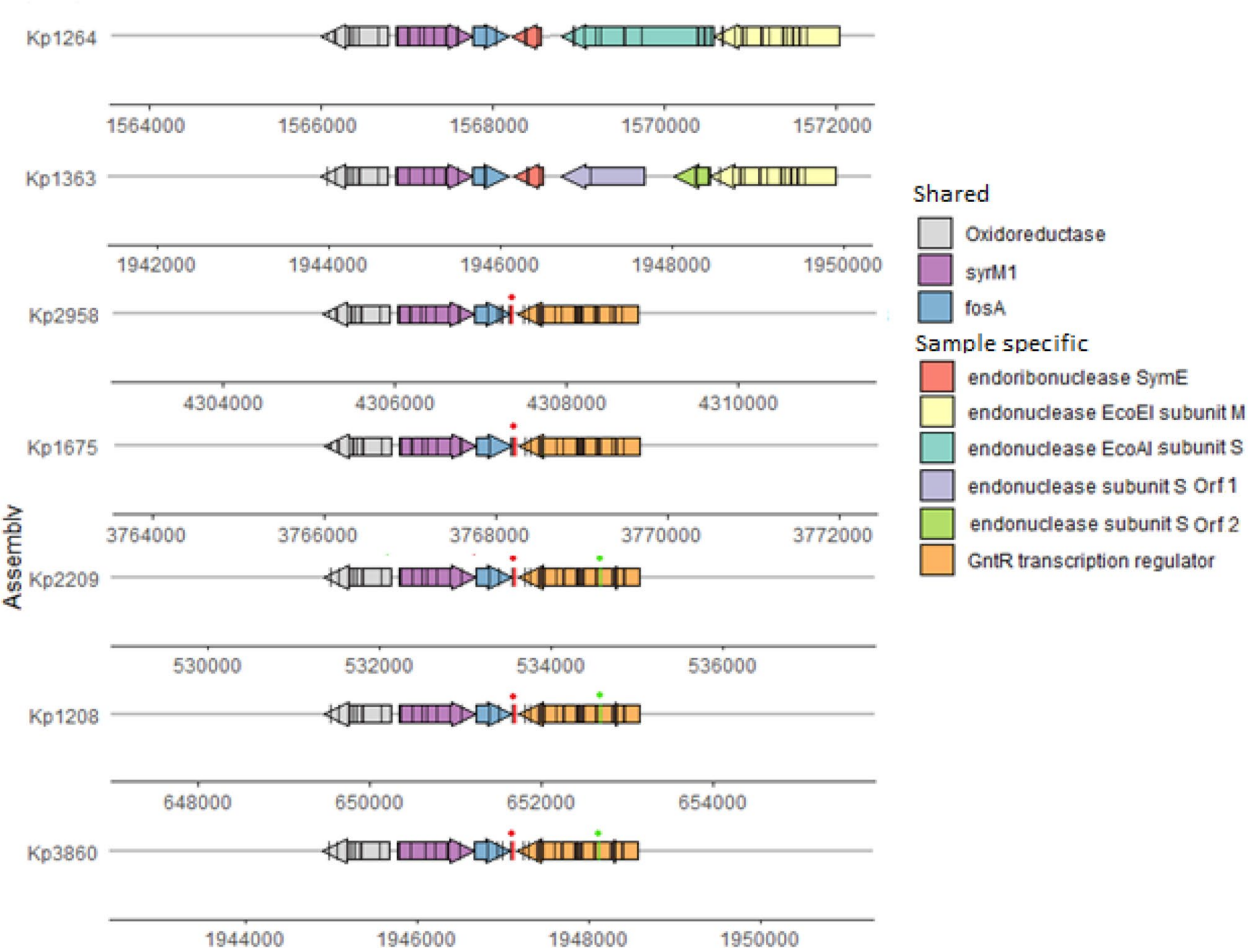
The *fosA* gene and surrounding region. (Black bars) methylated GATC motifs; (red bars with red dot above) unmethylated GATC motifs downstream of *fosA* on the same strand; (green bars with green dot above) unmethylated GATC on the opposite strand of GntR family transcription regulator coding sequence. Kp1264 and Kp1363 lack a GATC motif downstream of *fosA* due to an insertion of endonuclease. The other samples all have a single unmethylated GATC motif downstream of *fosA* on the same strand as *fosA* coding sequence. The only other unmethylated GATC in these regions are the motifs on GntR family transcription regulator.

Methylated motifs (see Table 2) covered a large portion of the genome and were relatively evenly distributed. Therefore, we focused on unmethylated motifs 50bps upstream and downstream of the genes. Upstream sequences capture gene promoters, whereas downstream sequences provide a control. We focused on the genes and motifs that have higher than expected number of unmethylated motifs (Table 3) in most isolates (> 3/7) (see Data S1 for the full list of identified genes). All such cases had a GATC motif, with only *fosA* and *tnpB* IS3 family (IS2 group) transposase genes having a consistently unmethylated motif downstream. *TnpB* is a key component of mobile genetic elements’ transposition mechanism, and in four samples that had the gene, it was located on a large (197–284kbp) IncFIB(B) plasmid containing various heavy metal and AMR loci. The *fosA* gene provides *Kp* with an inherent low-level resistance to the fosfomycin antibiotic^29,30^. Only one isolate (Kp1264) was resistant to fosfomycin (Table S1), but it was one of two isolates (Kp1264 and Kp1363; Fig. 3) that had the R–M system inserted after *fosA*. Both *fosA* and *tnpB* genes lacked methylation at a GATC motif downstream of the gene, so the unmethylated GATC should not be directly affecting promoter region; however, the lack of methylation may indicate that MTase cannot access the site.

**Table 3:** Genes which have an unmethylated GATC motif 50bps upstream/downstream in at least four samples.

Location	Product	RefSeq	GATC motif 50 bps up/down of gene is unmethylated							Binomial test p-value
			Kp1675	Kp1264	Kp1208	Kp1363	Kp2958	Kp2209	Kp3860	
Up	D-ribose transporter subunit RbsB	VEC00318.1	Yes	Yes	Yes	Yes	Yes	Yes	Yes	1.8E-12
Up	Branched-chain amino acid transport protein azlC	AHM82117.1	Yes	Yes	Yes	NM	Yes	Yes	Yes	8.6E-11
Up	D-arabinitol dehydrogenase	BAH64332.1	No	Yes	Yes	Yes	Yes	Yes	Yes	5.9E-10
Up	DnaK suppressor protein	BAH61791.1	Yes	No	Yes	Yes	Yes	Yes	Yes	5.9E-10
Up	BCCT family transporter	AHM80284.1	NM^a^	Yes	Yes	Yes	Yes	Yes	No	2.4E-08
Down	FosA family fosfomycin resistance glutathione transferase	QBH08895.1	Yes	NM	Yes	NM	Yes	Yes	Yes	4.1E-09
Down	IS3 family transposase	VEC38624.1	NM	NM	Yes	NM	Yes	Yes	Yes	1.9E-07
Up	Transcriptional regulators of sugar metabolism	VEC00015.1	No	Yes	Yes	NM	Yes	No	Yes	2.8E-06
Down	DUF1145 family protein	AHM77076.1	Yes	No	Yes	Yes	No	Yes	No	6.4E-06

Every sample has either one copy of the gene or no copies. The p-value null hypothesis is probability of gene’s GATC motif being unmethylated is the same as probability of any GATC motif being unmethylated. None of the non-GATC motifs had a p-value below 0.05.

NM = no motif.

^a^Missing start codon.

Of the seven isolates with high quality site methylation data, only Kp1264 (ST147) and Kp1363 (ST76) did not have the unmethylated motif downstream of *fosA*. In both isolates, the required GATC motif was missing due to insertion of an endonuclease gene (> 3 kbp) (Fig. 3). To assess the frequency of this insertion we analysed available *Kp* assemblies (n = 3584) from the NCBI database. We found that 99.4% of assemblies had the *syrM1* gene upstream of chromosomal *fosA*. The exceptions were 23 samples from diverse geographical regions and sequence types (Table S3). Further, we observed a limited variety of genes downstream on the opposite strand of *fosA* (Table 4). The top five genes accounted for 96.2% of the assemblies, with the predominant one being the GntR family transcription regulator (68.3%)^31^. For the ST147 sequence type (NCBI, n = 131), we found most assemblies (130/131) had toxic protein SymE in the same position. The outlying assembly (GCA_004145685.1_ASM414568v1_genomic) had a truncated version of SymE due to the scaffold ending in the coding sequence. For the ST76 sequence type, all NCBI isolates (n = 7) and Kp1363 (one locus mismatch) had coding sequence of endoribonuclease SymE downstream of *fosA* (Table 4).

**Table 4:** Genes and proteins encoded by coding sequences immediately downstream on opposite strand of chromosomal *fosA*.

*Gene* or product	ST258	ST11	ST15	ST147	ST405	ST101	ST231	Other	% of Total
GntR family transcriptional regulator	209	477	200				57	1408	68.3
Putative protein			1					306	8.6
DNA-dependent helicase II					23			289	8.1
5′-nucleotidase						88		130	6.1
Toxic protein SymE				130				56	5.2
endoribonuclease SymE								59	1.7
*yjiR_1*	1	3						18	0.7
*yjiR_2*	8	3	1					5	0.5
Hypothetical protein	1					6		7	0.4
Type I restriction endonuclease								6	0.2

Together these account for 99.6% of downstream elements in 3584 *K. pneumoniae* samples. For a given MLST, all samples normally have the same gene downstream of *fosA*. Genes *yjiR_1* and *yjiR_2* are truncated versions of the GntR family transcription regulator.

For a random subset of unmethylated motifs (from Table 3), we examined the IPDs of individual reads to evaluate the existence of distinct subpopulations with regard to the methylation status of genes, thereby investigating potential intra-populational epigenetic diversification (Figure S4). Standard SMRT Analysis output files report the methylated fraction of reads in the sample, but only for motifs that were called as methylated. We modified the SMRT Analysis algorithms to produce per base IPD values for all nucleotides. After analysing the underlying reads, we found that IPD levels in some genes, including *fosA* and *dksA* (DnaK suppressor protein), differentiated two subpopulations corresponding to methylated and unmethylated cells. By contrast, we observed no methylated subpopulation in *mglB* (D-ribose transporter subunit RbsB).

## Discussion

Across seven *Kp* isolates with high quality PacBio data, we identified the common Dam based methylation mechanism involving GATC motifs, as well as several type I R–M systems. With a type I R–M system, we observed that the abundance of recognition motifs did not vary between chromosomes of different assemblies, but that some depletion of recognition motifs has occurred in PEGEs. This observation supports the role of type I R–M system as a defence mechanism against invasion by foreign DNA, but not its regulatory function, as chromosomal abundance was not correlated to specificity subunit. In contrast, there was a clear difference in abundance of the GATC motif on chromosomes and PEGEs, which suggests that the motif has a function as identified by previous research^8,9^.

Because GATC motifs are known to have a regulatory function, we expected to find that some genes had consistently unmethylated GATC in gene promoter regions^8,9^. However, the finding of consistently unmethylated GATC downstream of genes (e.g. *fosA* and *tnpB*) is intriguing. This outcome may be an indication of a secondary structure that is inaccessible to MTase, or that the GATC motif downstream of *fosA* and *tnpB* is a distant promoter or regulatory region. The phenotypic impact of this GATC motif requires follow-up experiments ideally via single nucleotide site-directed mutagenesis as to prevent methylation with minimum downstream effects. Analysis of this region has also led us to identify the limited variety of recombination events around *fosA* and their potential MLST specificity. Toxic protein SymE is part of the plasmid toxin-antitoxin system. To assess its prevalence in *Kp* we evaluated 131 randomly chosen ST147 samples from the NCBI database. We found the same *fosA-SymE* sequence in all but one of them, but in no other major ST. The potential impact of the *SymE* insertion on fosfomycin resistance should be evaluated in follow-up experiments. The other coding sequences in that location do not appear to be part of the toxin-antitoxin system^32^, and therefore this locus has the potential to evaluate the robustness of a *Kp* phylogeny based on sequence types. We would expect that sequence types with the same insertion would cluster together, and the opposite observation would indicate possible recombination of MLST genes.

The general lack of resistance to fosfomycin in *Kp* species, despite decades of active use of fosfomycin for infection control^30,33^, is in contrast to the emergence of resistance to other antimicrobials. However, *Kp* has been reported to acquire resistance in vitro within 24 hours of exposure to fosfomycin^34^; though this resistance does not seem to persist as clinical studies report the limited spread of fosfomycin resistance^33^. The identified unmethylated GATC motif downstream of *fosA* may be correlated with the rapid acquisition of fosfomycin resistance in individual isolates. Our dataset is too small to draw any robust statistical inference, but the only sample in our study resistant to fosfomycin, Kp1264, is one of the two samples which lacks a GATC motif downstream of *fosA* (Fig. 3). The plasmid toxin-antitoxin system downstream of *fosA* in both Kp1363 and Kp1264 requires constant transcription, which means the genomic region is accessible to transcription machinery; whereas unmethylated GATC may be the result of DNA regional conformation inaccessible to Dam. It is unlikely that a lack of methylation downstream of the *fosA* gene is itself the fosfomycin resistance mechanism. If correlation exists, the more likely explanation is that conformation of the genomic region that prevents methylation, also prevents *fosA* transcription. This hypothesis is particularly relevant for the treatment of complicated infections by carbapenemase-producing strains, for which fosfomycin is often used in combination with another drug (e.g. colistin) as a last resort therapeutic option^35,36^.

Overall, our work has provided new insights into methylation and potential MLST specificity. We have generated eight new reference genomes for *Kp* and analysed their methylomes. The findings reinforce the role of type I motifs as a defence mechanism against foreign DNA. We also identified a higher than expected rates of the GATC motif on *Kp* chromosomes which, together with absence of respective endonuclease, support existence of Dam function such as DNA mismatch repair^37^ and gene expression regulation^9^. We identified two genes, *fosA* and *tnpB,* which have an unmethylated GATC motif downstream of each locus. Methylation analysis may be useful for identification of distant regulatory regions or frequent secondary DNA structures. In particular, a lack of methylation downstream of *fosA* genes could explain not only rapid emergence of resistance in individual samples, but also lack of widespread resistance in *Kp*. Further, using a bioinformatics approach we detected the presence of both methylated and unmethylated sequencing reads existing within individual samples, potentially representing subpopulations of distinct epigenetic lineages, which could contribute to stochastic phenotypic switching mechanisms in bacteria^38,39^. Such heterogeneity can be investigated in more depth using a SMALR approach^40^. This important biology and other findings should be evaluated in larger genomic and functional studies, and could lead to new insights into *Kp* infection control.

## Methods

### Sample collection, culture and sequencing

All eight samples were collected from Hospital de Santa Maria (n = 6) and Hospital St Antonio dos Capuchos (n = 2) in Lisbon, Portugal. The samples were cultured and tested for AMR as described previously^28^. DNA was extracted from strain cultures, grown overnight at 37ºC on Mueller–Hinton Agar. DNA extraction was carried out using the Cetyl trimethylammonium bromide (CTAB) method previously described using Tris–Acetate buffer (10 mM, pH 8)^41^. Our samples were generated without TET1 oxidation; thus we could not examine 5mC methylation^12,42^. All DNA samples were sequenced at the Genome Institute Singapore on the PacBio RSII platform.

PCR and Sanger sequencing-based MLST analysis was based on fragments of seven housekeeping genes: *rpoB* (beta-subunit of RNA polymerase), *gapA* (glyceraldehyde 3-phosphate dehydrogenase), *mdh* (malate dehydrogenase), *pgi* (phosphoglucoseisomerase), *phoE* (phosphorine E), *infB* (translation initiation factor 2), and *tonB* (periplasmic energy transducer). Details of the MLST scheme including amplification and sequencing primers, allele sequences and MLSTs are available on the Institute Pasteur’s MLST Web site (https://bigsdb.pasteur.fr/klebsiella/klebsiella.html).

### Bioinformatic analysis

A summary of the bioinformatic and analysis pipeline is provided (Figure S3). Data assembly was performed using three different software tools (HGAP3, Canu and Flye). HGAP3 is part of the SMRT Analysis toolkit (version 2.3), while Canu and Flye software are standalone^43–45^. Short read polishing was not performed due to > 100-fold long read coverage for each sample. We have aligned raw reads to the assemblies and examined region ± 2000 nt around *fosA* gene in each assembly. We did not observe^46^ any SNPs, InDels or alignment abnormalities. Between samples, the coverage of the region ranged from 77- to 219-fold. Generated assemblies were compared using the Busco enterobacteriales (odb10) dataset^47^. For each sample the assembly with highest Busco complete gene count was selected. The sum of complete and fragmented genes yielded the same “best” assemblies. While the HGAP3 assembly had marginally higher N50 score, in most cases its Busco scores were substantially lower. All but one of the selected assemblies had a scaffold longer than 5Mbp. The outlying assembly, Kp1675, had a chromosome split into two scaffolds which was considered an acceptable trade-off for a 5.3% higher share of complete genes. The in silico resistance profile, MLST, O-locus and K-locus types were determined using the kleborate tool^48,49^. Plasmid and Insertion Sequence (IS) identities were established using PlasmidFinder^50^ and ISFinder^51^.

### Methylation analysis

We used the generated assemblies to perform methylation detection by applying the PacBio SMRT Analysis toolkit (v2.3). To discriminate between natural variation in IPD and true methylation the toolkit relies on expected distribution of unmethylated IPDs given the nucleotide sequence context. When IPD value exceeds the probability threshold, we used default p-value 0.01, the relevant base is called as modified. The calculations are implemented in *KineticWorker.py*, which is part of SMRT Analysis toolkit^52^. After comparing the generality of motifs versus the frequency of motif methylation for each isolate, we removed the motifs for which less than 60% of motif occurrences were methylated. We retained two motifs for the Kp2564 isolate which had methylation fractions below 60% (Table 2), where the low level of methylation was result of insufficient sequencing coverage.

### NCBI datasets

To assess the abundance of GATC motif on *Kp* chromosomes, we downloaded from the NCBI RefSeq database all *Kp* assemblies where their longest chromosomal contig was at least 4.5Mbps (n = 673)^53^. For an analysis of the prevalence of GATC motif downstream of *fosA* gene, we downloaded all *Kp* assemblies in the NCBI pathogen database (as of November 15, 2019; n = 3584)^21^. For reconstruction of a *Kp* phylogenetic tree we determined the MLST^20^ of each of the 3584 assemblies using kleborate software^48^. For each MLST which had more than nine samples from different geographical locations (city, region, or country) and year of collection data, we included a single randomly selected assembly for phylogenetic reconstruction. A total of 83 isolates were used in the construction of the phylogenetic tree. The tree was constructed using IQTREE software^54^ with the GTR + F + G4 model.

### Ethical statement

The study was approved by the Hospital de Santa Maria and Hospital St Antonio dos Capuchos Ethics Committees, and all methods were performed in accordance with the relevant guidelines and regulations, including informed consent from all patients.

## Supplementary Information

Supplementary Information 1.

Supplementary Information 2.

## Data Availability

The assembled sequences are deposited in the European Nucleotide Archive (project PRJEB38289). Accession numbers and metadata are presented in Table S1. The authors confirm all supporting data, code and protocols have been provided within the article or through supplementary files.
