# Supplementary material for: Methylation analysis of Klebsiella pneumoniae from Portuguese hospitals
Source: Sci Rep. 2021 Mar 22;11:6491. doi: 10.1038/s41598-021-85724-2 (PMC7985491; doi:10.1038/s41598-021-85724-2)
Supplement: Supplementary file 2 — Supplementary Information 2. [file 41598_2021_85724_MOESM2_ESM.docx]

# **Title: Methylation analysis of *Klebsiella pneumoniae* from Portuguese hospitals**

**Short Title: The *Klebsiella pneumoniae* methylome**

# Anton Spadar^1^, João Perdigão^2^, Jody Phelan^1^, James Charleston^1^, Ana Modesto^2^, Rita Elias^2^,

# Paola Florez de Sessions ^3^, Martin L. Hibberd ^1^, Susana Campino^1^, Aida Duarte ^4,5^, Taane G. Clark ^1,6^

**Figure S1**

**The motifs analysed.** Comparison between the generality of all methylation motifs identified by SMRT Analysis and the rate of motif methylation. Only motifs with >60% share of methylation were selected for further analysis. The motifs that cover >100% of chromosome were very general non-palindromic motifs. The total length of some motifs exceeds the chromosome length due to occurrence on both DNA strands. The three analysed motifs with a share of methylation below 50% are GATC and both partners of GCAYN_5_GTT (from Kp2564). The low methylation rate is due to low sequencing coverage.

**Figure S2**

**GATC motifs inter-pulse duration (IPD) ratio for the fosA centred chromosomal regions** (see **Figure 3**). The four outliers, representing unmethylated GATC downstream of fosA are visible at the bottom of the whiskers for Kp1675, Kp2209, Kp2958 and Kp3860. The single outlier of Kp1208 is less visible. The two samples without outliers Kp1363 and Kp1264 do not have the GATC motif downstream of fosA due to an insertion of endonuclease (see **Figure 3**).


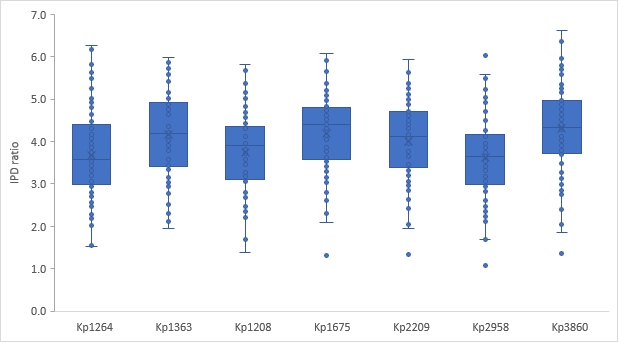


**Figure S3**

**The analytical pipeline**


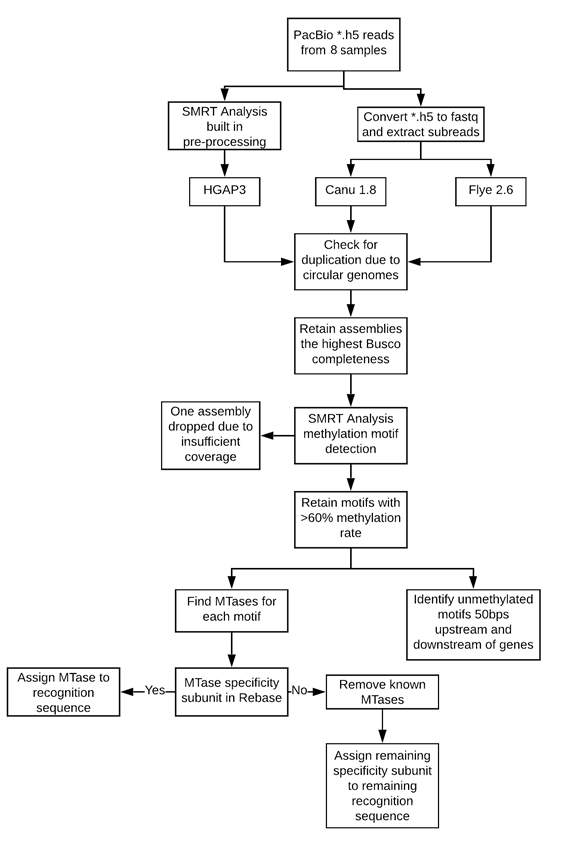


**Figure S4. Log_10_(IPD ratios) around GATC motifs upstream of (a) dksA (BAH61791.1) and (b)** **mglB (VEC00318.1) genes in the Kp3860 isolate.** Red is adenine in GATC motif. The difference between methylated and unmethylated calls in dksA **(a)** is not large and is driven by different means. Some outliers are also visible, and some are hidden by long whiskers. By contrast, in mglB **(b)** adenine does not stand out from other bases.

**(a)**

**
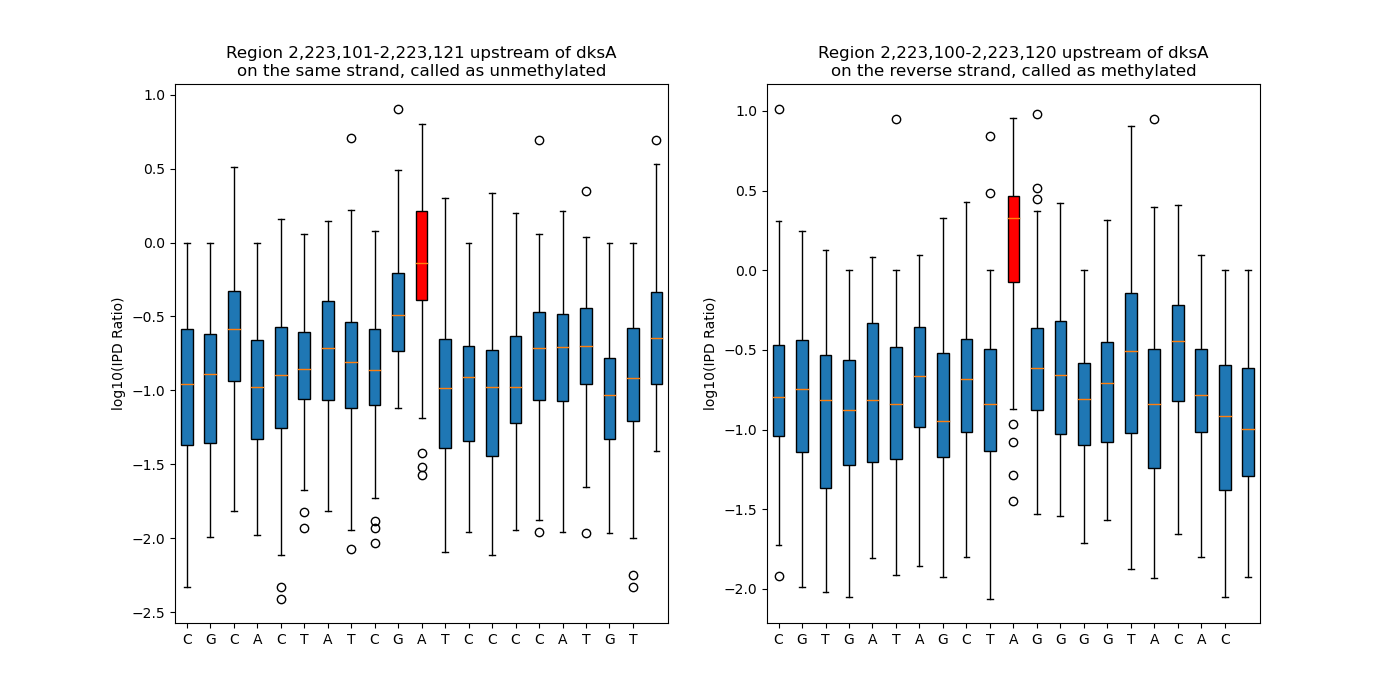
**

**(b)**

**
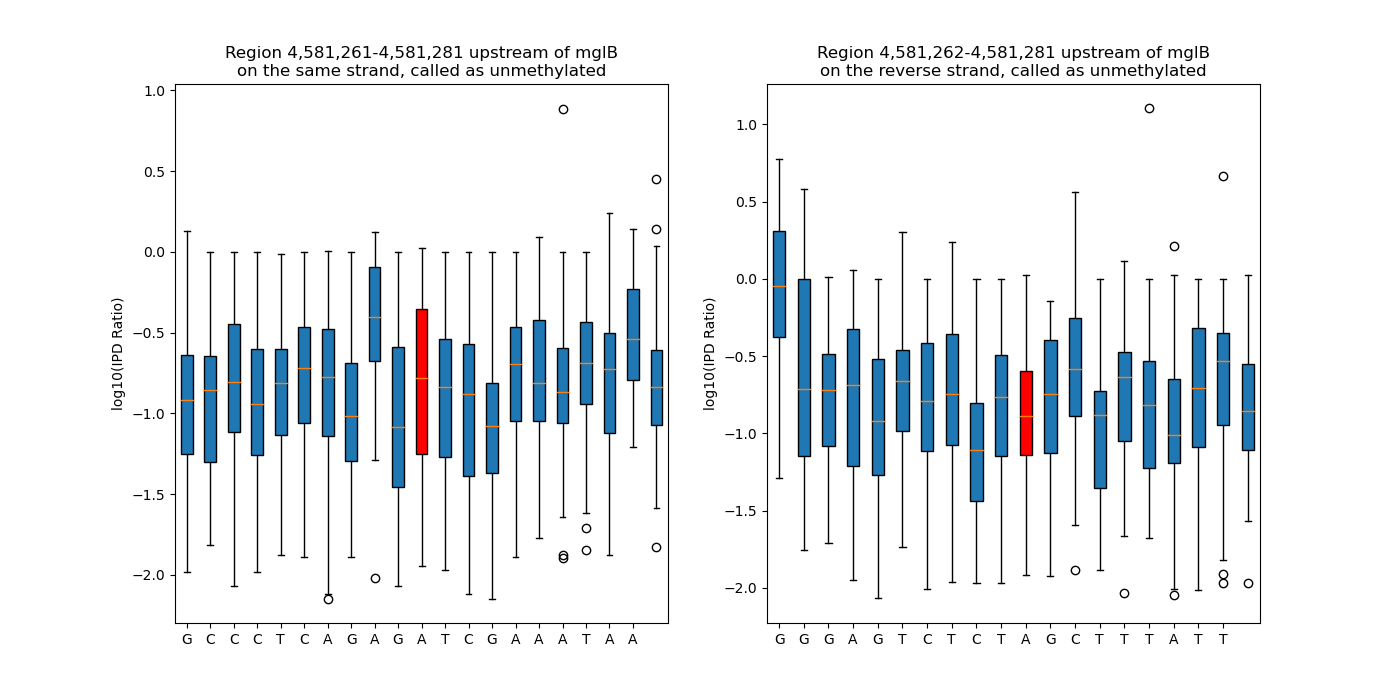
**

**Table S1**

**Antimicrobial Resistance**

| ID | NCBI accession | CTX-M-15 | OXA-1 | KPC-3 | AMC | FOX | CTX | CAZ | IPM | GM | CIP | FOS |
| --- | --- | --- | --- | --- | --- | --- | --- | --- | --- | --- | --- | --- |
| Kp1363 | CAESWS000000000.1 |  |  |  | S | S | S | S | S | S | S | S |
| Kp1208 | CAESWW000000000.1 |  |  |  | R | S | S | R | S | R | S | S |
| Kp1264 | CAESWT000000000.1 | Tn3  IncFIA(HI1) | Tn3  IncFIA(HI1) |  | R | R | R | R | S | R | R | R |
| Kp1675 | CAESWY000000000.1 | Tn3 | Tn3 |  | R | S | R | R | S | R | S | S |
| Kp2209 | CAESWV000000000.1 | IncFIB(K) |  |  | R | S | R | R | S | S | S | S |
| Kp2564 | CAESWU000000000.1 |  |  | IncFIA | R | R | R | R | R | R | R | S |
| Kp2958 | CAESWX000000000.1 |  |  |  | R | R | R | R | I | R | R | S |
| Kp3860 | CAESWZ000000000.1 | IncFIB(K) | IncFIB(K) |  | R | S | R | R | S | R | R | S |

S = sensitive, R = resistant, AMC= amoxicillin + clavulanic acid, FOX = cefoxitin, CTX = cefotaxime, CAZ = ceftazidime, IPM = imipenem, GM = gentamicin, CIP = ciprofloxacin, FOS = fosfomycin

**Table S2. Comparison of abundance ratio (AR) of recognition motifs on chromosomes (contigs >1Mbp) and putative extra-chromosomal genetic elements (PEGEs; contigs <1Mbp).**

| **Group 1** | **Group 2** | **Mean AR % Group 1** | **Mean AR % Group 2** | **Wilcoxon rank sum test P-value** |
| --- | --- | --- | --- | --- |
| Chromosome type I recognition motifs native to assembly | Chromosome type I recognition motifs from other assemblies | 0.192 | 0.234 | 0.515 |
| PEGE type I recognition motifs native to assembly | PEGE type I recognition motifs from other assemblies | 0.183 | 0.268 | 0.187 |
| Chromosome type I recognition motifs | PEGE elements (>10kbp) type I recognition motifs | 0.230 | 0.262 | 0.234 |
| GATC motifs on chromosome | GATC motifs on PEGEs | 2.25 | 1.45 | 0.000003 |

**Table S3. Of the 3,584 K. pneumoniae genomes analysed, only these 23 did not have gene syrM1 immediately upstream of fosA gene.**

| **Assembly ID** | **Sequence type** | **Country** |
| --- | --- | --- |
| GCA_000529745.1 | ST3695 | Austria |
| GCA_000529945.1 | ST15-3LV | Austria |
| GCA_000529425.1 | ST3827-3LV | Austria |
| GCA_003095515.1 | ST258 | Brazil |
| GCA_004127575.1 | ST256 | China |
| GCA_002173825.1 | ST25 | China |
| GCA_900516965.1 | ST15 | Hungary |
| GCA_004145895.1 | ST231 | India |
| GCA_900181455.1 | ST48 | Pakistan |
| GCA_001316495.2 | ST101 | South Africa |
| GCA_001316565.3 | ST101 | South Africa |
| GCA_001316645.2 | ST101 | South Africa |
| GCA_001316785.2 | ST101 | South Africa: |
| GCA_001316895.2 | ST101 | South Africa |
| GCA_001316985.2 | ST101 | South Africa |
| GCA_002510005.1 | ST3800 | South Africa |
| GCA_002510215.1 | ST1552 | South Africa |
| GCA_002522955.1 | ST101 | South Africa |
| GCA_002856415.1 | ST199 | USA |
| GCA_002856565.1 | ST133 | USA |
| GCA_002856885.1 | ST968-4LV | USA |
| GCA_002857345.1 | ST298 | USA |
| GCA_001066585.1 | ST3602 | USA |
